# Hourly global horizontal irradiance data of three stations in Punjab, Pakistan

**DOI:** 10.1016/j.dib.2021.107371

**Published:** 2021-09-17

**Authors:** Zia ul Rehman Tahir, Muhammad Asim, Muhammad Azhar, Ghulam Murtaza Amjad, Muhammad Junaid Ali

**Affiliations:** Department of Mechanical Engineering, University of Engineering and Technology Lahore, Pakistan

**Keywords:** Solar radiation, Global horizontal irradiance, Clearness index, Bias correction methods, CFSR, MERRA-2, ERA-5, Pakistan

## Abstract

This paper presents hourly Global Horizontal Irradiance (GHI) measured data at three stations (Lahore, Multan, Bahawalpur) in Punjab, Pakistan. The estimated GHI data from three reanalysis datasets have also been presented. Clearness index (*K_T_*), Solar zenith angle (θ_sza_) and Periodicity factor (*Pf*) were calculated and used to develop bias correction models. The estimated corrected GHI data using best model M20 for years 2015 and 2016 is also presented.

## Specifications Table

**Table:** 

Subject	Renewable Energy
Specific subject area	Solar Energy Resource Assessment
Type of data	Tables, Figures
How data were acquired	GHI data was measured using Kipp and Zonen pyranometers, CMP10 and CMP21
Data format	Raw data and analysed data
Description of Raw data collection	Energy Sector Management and Assistance Program (ESMAP) of the World Bank measured solar radiations data
Parameters for data collection	ESMAP assessed quality of measured data. There were no gaps in surface measured data. Reliability of the measured data was ensured by applying the quality checks developed by Baseline Surface Radiation Network (BSRN).
Data source location	Country: Pakistan Institution: ESMAP Stations Coordinates (Latitude × Longitude): Bahawalpur: 29.325° × 71.819° Lahore: 31.694° × 74.244° Multan: 30.165° × 71.498°
Data accessibility	Data is included in this article
Related research article	Zia ul Rehman Tahir, Muhammad Asim, Muhammad Azhar, Muhammad Amjad, Muhammad Farooq, Muhammad Junaid Ali, Syed Uzair Ahmad, Ghulam Murtaza Amjad, Afkar Hussain, Improving the Accuracy of Solar Radiation Estimation from Reanalysis Datasets using Surface Measurements, Journal of Sustainable Energy Technologies and Assessments, https://doi.org/10.1016/j.seta.2021.101485

## Value of the Data

• The data provided in this article can be used for solar research in Pakistan, specifically in Punjab.
• The data is useful in site selection based on GHI potential in relevant region.
• The raw data can be utilized for estimation and validation of bias correction irradiance models.
• Findings from the data bring awareness for establishment of commercial solar PV plants.
• Findings from bias corrected data can be used for developing multi decadal time series of these stations to develop economic and technical feasibility of future projects.

## 1 Data Description

**Fig. 1:**
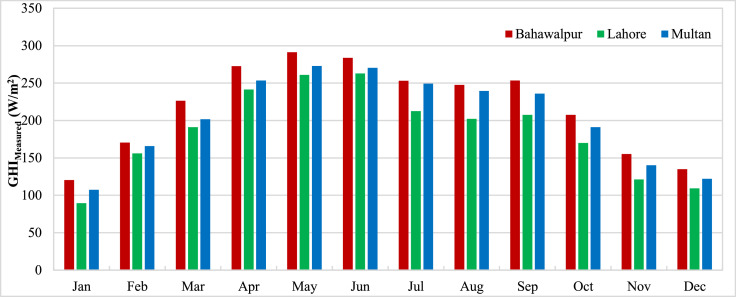
Monthly mean measured GHI for Bahawalpur, Lahore and Multan.

**Fig. 2:**
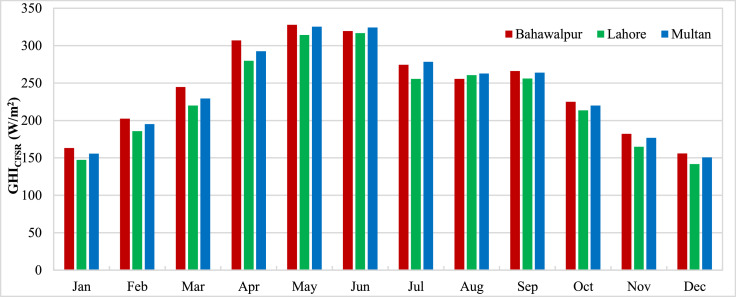
Monthly mean estimated GHI from CFSR for Bahawalpur, Lahore and Multan.

**Fig. 3:**
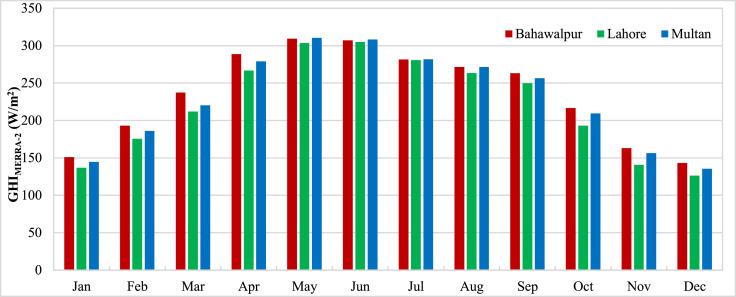
Monthly mean estimated GHI from MERRA-2 for Bahawalpur, Lahore and Multan.

**Fig. 4:**
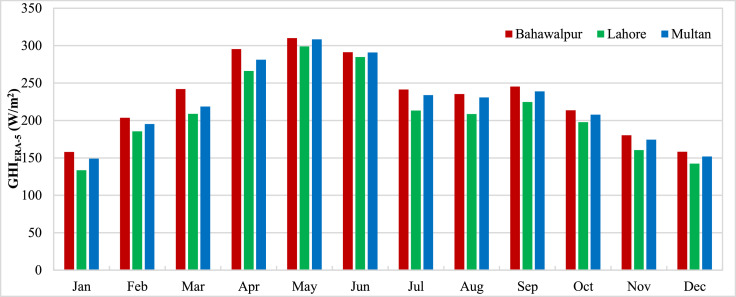
Monthly mean estimated GHI from ERA-5 for Bahawalpur, Lahore and Multan.

**Fig. 5:**
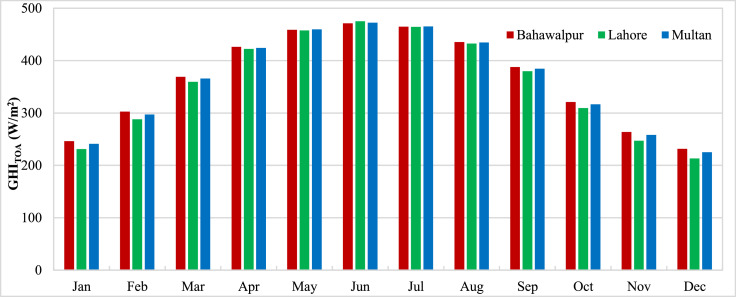
Monthly mean GHI at top of atmosphere for Bahawalpur, Lahore and Multan.

**Fig. 6:**
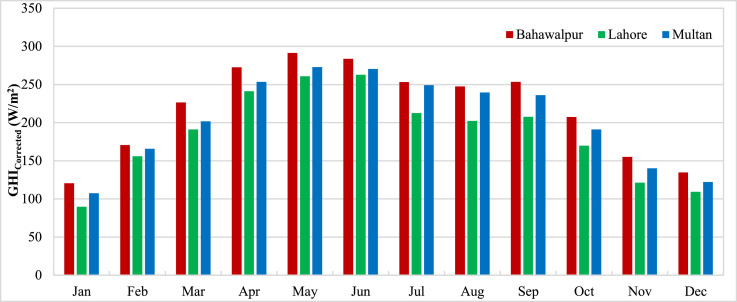
Monthly mean corrected estimated GHI using three datasets for Bahawalpur, Lahore and Multan.

The data presented in the article is measurements from three stations in Pakistan carried out by ESMAP of the World Bank. Three measurement stations were Bahawalpur, Lahore and Multan. The measurements for these stations were performed from 1st November 2014 to 30th April 2017. Hourly measurements of Global Horizontal Irradiance (GHI_Measured_) were used to calculate the monthly mean GHI for three stations and presented in Fig. 1. The monthly means show the variation of GHI due to seasonal anomalies over the year. Reanalysis data for GHI estimations from CFSR (GHI_CFSR_), MERRA-2 (GHI_MERRA-2_) and ERA-5 (GHI_ERA-5_) was accessed to assess the accuracy of estimates. Monthly means calculated from hourly estimates for CFSR, MERRA-2 and ERA-5 have been presented for three stations in Figs. 2, 3 and 4 respectively. The hourly GHI data at top of the atmosphere (GHI_TOA_) was calculated and monthly mean data is presented in Fig. 5. The reanalysis estimated GHI was corrected using best bias correction model and corrected monthly mean GHI (GHI_Corrected_) data for three stations is presented in Fig. 6.

## 2 Experimental Design, Materials and Methods

The GHI data at three stations was measured by Tier 1 (Bahawalpur) and Tier 2 (Lahore and Multan) measurement systems. Tier 1 measurement system was equipped with Kipp & Zonen CMP21 Pyranometer having CVF4 ventilation unit. Tier 2 measurement system was equipped with CSP Services Twin-Sensor Rotating Shadowband Irradiometer (RSI) and Kipp & Zonen CMP10 Pyranometer. The sampling rate of data was 1 Hz which was averaged to 10 min.

Tier 1 station measurements were performed at well-maintained location with grid connected electricity. GHI measurements were performed with ISO 9060 secondary standard pyranometer was mounted on automatic sun tracking system. Data transfer performed via GPRS was based on BSRN (Baseline Surface Radiation Network) standards. Tier 2 station measurements systems were installed at remote stations with self-sufficient operation and uncertainty.

The quality of measured raw data was checked using method proposed by the Baseline Surface Radiation Network (BSRN), the values lying outside the limits were flagged and replaced with zero. The cosine error is a function of solar zenith angle (θ_sza_), which is maximum at lower solar altitude, so solar radiation data corresponding to θ_sza_ > 85° was not considered.

The hourly clearness index (the ratio of GHI_Measured_ and GHI_TOA_) was calculated and GHI_TOA_ was calculated using the Eq. (1.10.4) of Ref. [1]. The value of *K_T_* shows the atmospheric transparency and incorporates the effect of atmospheric pollutants, aerosols and clouds. Clearness index depends on solar zenith angle which is a function of latitude, day and time. The modified clearness index (*K_T′_*) to avoid the effect of solar zenith angle in estimations of sky clearness was calculated using equation proposed by Perez et al. [2].

### 2.1 Bias correction of estimated GHI

The corrected GHI is of use for region with limited availability of high-quality measured data and the unavailability of long-term data. The available reanalysis products provide GHI estimates with some uncertainties especially under cloudy sky conditions. The estimated GHI was corrected using 20 models, details of these model can be found in Ref. [3], the model M20 was best model to correct estimated data, the estimated data corrected by M20 model (GHI_Corrected_) is also presented. The model M20 is presented, *K_Tc_* is corrected clearness index, µ is cosine of solar zenith angle and *Pf* is periodicity factor. The *K_T1_, K_T2_* and *K_T3_*, are clearness indices form reanalysis datasets CFSR, ERA-5 and MERRA-2 respectively.

$$\begin{array}{ccc} M20:{K_{T}}_{c} & = & a_{20}+b_{20}K_{T1}+c_{20}K_{T2}+d_{20}K_{T3}+e_{20}\mu K_{T1}+f_{20}\mu K_{T2}+g_{20}\mu K_{T3}+h_{20}\mu \\ & & +\;i_{20}\text{cos}\left(\mathrm{Pf}\right)+j_{20}\text{sin}\left(\mathrm{Pf}\right) \end{array}$$

## CRediT authorship contribution statement

**Zia ul Rehman Tahir:** Conceptualization, Methodology, Supervision. **Muhammad Asim:** Validation, Formal analysis. **Muhammad Azhar:** Methodology, Writing – original draft, Writing – review & editing. **Ghulam Murtaza Amjad:** Writing – original draft, Investigation. **Muhammad Junaid Ali:** Formal analysis, Writing – review & editing.

## Declaration of Competing Interest

The authors declare that they have no known competing financial interests or personal relationships that could have appeared to influence the work reported in this paper.

## Appendix Supplementary materials

Image, application 1
